# Impact of the new guidelines of the American Thyroid Association on the treatment of the differentiated thyroid tumor in an Italian center with medium-high volume thyroid surgery

**DOI:** 10.1186/s12893-018-0462-8

**Published:** 2019-04-24

**Authors:** Aldo Bove, Paolo Panaccio, Gino Palone, Ludovica Esposito, Lucia Marino, Giuseppe Bongarzoni

**Affiliations:** Department of Medicine, Dentistry and Biotechnology – University “G. D’Annunzio”, Via dei Vestini, 66100 Chieti, Scalo Italy

**Keywords:** Differentiated thyroid cancer, Thyroid surgery, Total thyroidectomy, Lobohystmectomy, American Thyroid Association guidelines task force

## Abstract

**Background:**

The therapy for differentiated thyroid tumors is currently built upon two cornerstones: the stage of the disease and the new guidelines of the American Thyroid Association, jointly converging to lobohystmectomy for selected cases that meet certain criteria. The aim of the study was to relate these guidelines to the activity of an Italian center with a medium-high volume of thyroidectomies in a region with a high rate of endemic disease of the thyroid.

**Methods:**

In order to conduct the analysis, the clinical records of the last 3 years, including 194 cases of total thyroidectomy and 3 lobohystmectomy, were taken into consideration. There were 46 cases of differentiated thyroid cancer (18 incidental tumors were found during thyroidectomies for benign diseases). Postoperative complications, patient characteristics and the stage of the tumor were assessed in relation to the new ATA guidelines.

**Results:**

All patients underwent total thyroidectomy, with 2 of them also undergoing lymphadenectomy. The incidence of transient hypoparathyroidism was 19% with 1 case of permanent deficit. No cases of recurrent nerve injury were reported. Twenty-five out of the 28 patients with cancer preoperatively diagnosed were found with more nodules and in 15 of them the nodule had a diameter bigger than 1 cm. All the parameters suggested lobohystmectomy only for one case.

The treatment for the differentiated thyroid tumor is still widely discussed. Above all, differences between populations, screening methods and surveillance programs are still evident.

**Conclusions:**

The ATA guidelines applied to our cases, even if limited, have shown limited applicability to our study, mainly due to the high incidence of multinodularity and the size of the nodule: typical characteristics of a region with a high rate of endemic thyroid pathology.

## Background

The diagnosis of thyroid nodules has increased in the last decade and, although most of them turned out to be benign, between 7 and 15% of the cases could present differentiated malignant neoplasms [1]. The increase in the incidence of differentiated thyroid tumors is largely due to overdiagnosis resulting from ultrasound screening and from cytological examination (needle aspiration) but also in part to a greater exposure to radiating agents [2].

Luckily, the prognosis of these tumors is excellent with a survival percentage ranging between 98 and 95% [3]. Despite patients had previously been treated with total thyroidectomy, the new guidelines of the American Thyroid Association (ATA), published in January 2016, indicated the lobohystmectomy, for selected cases, as the best alternative, but also as a less invasive intervention [4]. This choice was recommended to limit the main complications of total thyroidectomy such as hypoparathyroidism and lesions of the inferior laryngeal nerve.

To this extent, peculiar characteristics of the nodule have been identified. Among those the most influential resulted to be: size, uniqueness, non-invasion of the capsule and absence of extraglandular disease, age inferior to 40 years, and non-exposure to radiant therapies, for which a loboistmectomy with the same distance results of total thyroidectomy is suggested.

It should be noted, however, that there are other guidelines in the literature [5] and numerous different suggestions, up to the hypothesis of just active surveillance [6]. This plethora of opinions leads us to think that the treatment of the differentiated thyroid tumor has not reached a unanimous consensus yet. These guidelines, even if discussed by international experts, may not be completely applicable to different cases of endemic thyroid diseases that often overlap with differentiated thyroid neoplasms [7]. Up until the recent past, our suggestion in the treatment of the differentiated thyroid tumor was the total thyroidectomy with lymphadenectomy of the central and latero-cervical areas, only in cases of positivity to clinical-instrumental preoperative work-up.

The aim of the study was to evaluate the impact of the new ATA guidelines in the indication of the type of surgery for the differentiated thyroid neoplasms in an Italian center with a medium to high number of thyroid surgeries.

## Methods

The study was based on thyroid surgery cases taken from January 2014 to December 2016. We propose total thyroidectomy also for the diffuse benign pathology such as multinodular goiter and M. of Graves. In addition to limiting the re-operation cases, total thyroidectomy allows a better management of thyroid function. Certainly, this must come whit a minimal incidence of complications. In our experience we found a rate of RNL palsy of 1.2% and a percentage of definitive hypocalcaemia of 2.8%. These data allow us to suggest total thyroidectomy also for benign pathology.

During this period, 194 total thyroidectomies and 3 lobohystmectomies were performed. In 46 patients (22.5% cases) the diagnosis of differentiated thyroid carcinoma was done (42 papillary, 2 follicular carcinomas, and 2 Hurtle cell carcinomas). Thirty-four women and 12 men, average age 48 aa (21–78).

In 10 cases, the diagnosis was pre-operative, in 18 cases there was a suspected preoperative cytology confirmed by surgery (subgroup A), and in 18 cases we found incidental microcarcinoma on thyroidectomy for benign disease (subgroup B). Italian consensus for the classification and reporting of thyroid cytology (ICCRTC) has been used.

Ten out of 18 patients that underwent FNAB were diagnosed as Tir 4, 8 as Tir3 B and 2 as Tir3 A.

In our experience the surgical positive matching percentage was 98% in Tir 4 and 55% in Tir3 B, while in the Tir3A group only 25% had a positive matching response after surgery.

We analyzed all the complications, (hypocalcemias, recurrent nerve paralysis, haemorrhages) the patient’s individual characteristics and the staging of the disease, and we relate them to the new ATA guidelines in order to identify possible cases that, according to these guidelines, could be treated with a lobohystmectomy.

## Results

All patients underwent total thyroidectomy. For 2 patients, central and latero-cervical lymphadenectomy was necessary. Results show zero mortality, a percentage of transient hypoparathyroidism around 19% (6 out of 46 patients), no lesions of the inferior laryngeal nerve but 1 case of definitive hypoparathyroidism (patient undergoing lymphadenectomy). There was also 1 case of post-operative hematoma that required an additional surgery (Table 1).

**Table 1:** Demographics data and complications after thyroidectomy

All patients (2014–2016)	197
Age *(years)* (range)	48 (21–78)
Male/female ratio	12/34
Transient Hypoparathyroidism	6/46 (19%)
Permanent Hypoparathyroidism	1/46 (2.2%)
Recurrent laryngeal nerve injury	0/46 (0%)
Post-operative haemorrhage	1/46 (2.2%)

Analyzing the data of the 46 patients and considering the criteria of the new ATA guidelines we reported nodule size inferior to 1 cm for 13 cases (46.4%), single nodule for 4 cases (7%), age inferior to 40 years aa for 5 cases (15%), non-invasion of the capsule for 44 cases (90%), no lymph node metastasis for 44 patients (90%). No patient reported exposure to radiant therapies. Post-surgery results turned out to present interesting features in cases of suspected preoperative diagnosis for differentiated thyroid carcinoma. The final histological check-up showed that 4 (8.7%) of these patients had a multifocal tumor (Table 2).

**Table 2:** Pathological findings

All patients (2014–2016)	197
Total thyroidectomy	194
Lobohystmectomy	3
Neoplastic disease	46/197 (23.3%)
- Preoperative malignant cytology	28/46 (60.9%)
- Incidental malignant disease	18/46 (39.1%)
Hystotype	
- Papillary carcinoma	42/46 (91.4%)
- Follicular carcinoma	2/46 (4.3%)
- Hurtle cells carcinoma	2/46 (4.3%)
Multifocal neoplastic disease	4/46 (8.7%)
Benign disease	151 (76.7%)

In the patients group with positive or indeterminate cytology, 25 out of 28 cases presented multinodular pathology, 24 were older than 40 years, 15 had a nodule with diameters bigger than 1 cm, 2 showed capsular invasion, 2 had lymph node metastasis and only 1 case (3.6%) responded to all the ATA parameters for a lobohystmectomy (Table 3).

**Table 3:** Histopathological characteristics according to ATA guidelines in the subgroup patients with pre-operative indeterminate or positive cytology for malignant disease

All patients (2014–2016)	28/197 (14.2%)
Nodules ≤1 cm	13/28 (46.4%)
Solitary nodule	3/28 (10.7%)
Age < 40 y	4/28 (14.3%)
No capsule invasion	26/28 (92.9%)
No metastatic lymphadenopaties	26/28 (92.8%)
Radiant therapy	0/28 (0%)
Patients satisfying all ATA parameters for Istmo-lobectomy	1/28 (3.6%)

## Discussion

In the last decade, the therapy for differentiated thyroid tumor underwent a reverse trend; whereas in the past it was characterized by an aggressive approach, it is now modeled upon the stage of the disease [8].

Total thyroidectomy was supposed to definitively solve the clinical problem with a biological legitimation, removing the frequent plurifocality of the neoplasia [9]. Moreover, it led to a much-improved follow-up to a possible radiometabolic treatment. However, side effects included definitive hypoparathyroidism and lesions of the inferior laryngeal nerve [10].

Furthermore, these tumors often presented biological traits such as non-progressive nature and with a high percentage of long term survival. Therefore, more and more data confirmed the possibility of treating this type of tumor with a less aggressive intervention of lobohystmectomy [11].

According to the above-mentioned reasons, the ATA has changed its guidelines, which in the past always required a total thyroidectomy, but from January 2016 suggested the intervention of loboistmectomy in selected cases. The goal of this study was to evaluate the impact of these new guidelines in an Italian center with a medium to high thyroid surgery volume. It is necessary to have completed at least 30 total thyroidectomy (TT) surgeries per year to be included into this category [12]. Subject of this study were cases taken from the last 3 years in an area with a high rate of endemic goiter. It is important to remember that, in our case, TT had been offered for several years for benign pathology which has proved to be the best therapeutic choice for multinodular goiters [13] or M. of Graves [14].

First, the percentage of tumors was high, mainly influenced by the occurrence of incidental carcinomas (about 39.1%) discovered after surgery for initial benign pathology. For cases of preoperative or suspected diagnosis confirmed with surgery, the clinical data allowed to identify only 1 case (3.6%) that meets the criteria of the ATA guidelines to perform a lobohystmectomy.

In particular, there was a plurinodularity for 25 out of 28 patients with an expression of endemic thyroid disease. The problem of the possible plurifocality of the neoplasia remains unsolved; presence of contralateral malignant nodules, diagnosed as benign during pre-operative investigations, was reported for 28.9% of cases [15], while in long-term check-ups the risk of contralateral recurrence is reported about 4% [16]. Our study has shown that the incidence of plurifocality of the neoplasm is high (8.7%) even if lesions often have dimensions of at least less than 5 mm.

Even the size of the nodule for more than a half of our cases exceeded the diameter of 1 cm. Not everyone agrees, however, with this restriction; the British Thyroid Association suggests lobohystmectomy with nodules up to 4 cm [17] in size whereas others report excellent results even with nodules of 5 cm in size [18].

On the other hand, there is unanimous consensus in considering capsular invasion or lymph node metastasis as risk-factors for recurrence, therefore they should be treated with a TT surgery [19].

There are also several reports especially on Asian populations, which show a non-surgical attitude towards differentiated thyroid neoplasms with a possibility of active surveillance of the neoplasm and a low urgency for surgical intervention [20]. Taken into consideration are single-nodule patients who can be carefully followed for several years.

## Conclusions

A general treatment for the differentiated thyroid tumor has yet to find concordant opinions. Above all, differences between populations and screening methods and surveillance programs are still evident. The guidelines of ATA compared to our study, although with a small number of cases, have shown a low percentage of applicability, especially for the high incidence of multinodularity and for the size of the nodule, characteristic of a region with a high rate of endemic thyroid disease.

## Abbreviations

ATA: American Thyroid Association
TT: Total Thyroidectomy
